# hACE2 Fc-neutralization antibody cocktail provides synergistic protection against SARS-CoV-2 and its spike RBD variants

**DOI:** 10.1038/s41421-021-00293-y

**Published:** 2021-07-20

**Authors:** Junli Liu, Qi Chen, Shumin Yang, Ying Li, Yang Dou, Yong-Qiang Deng, Jinpeng Bi, Yongcong Tan, Hongfan Wang, Wei Gong, Xiaoyu Xu, Zhenhu Li, Guojun Lang, Cheng-Feng Qin, Bai Lu, Weidong Jiang

**Affiliations:** 1 Shanghai Henlius Biotech, Inc., Shanghai, China; 2 State Key Laboratory of Pathogen and Biosecurity, Beijing Institute of Microbiology and Epidemiology, AMMS, Beijing, China; 3 School of Pharmaceutical Sciences, IDG/McGovern Institute for Brain Research, Tsinghua University, Beijing, China; 4 Vazyme Biotech Co., Ltd., Nanjing, China; 5 Sanyou Biopharmaceuticals Co., Ltd., Shanghai, China; 6 Beijing Tiantan Hospital, Advanced Innovation Center for Human Brain Protection, Capital Medical University, Beijing, China

**Keywords:** Immunology, Cell biology

Dear Editor,

The COVID-19 pandemic caused by the new coronavirus SARS-CoV-2 has become a global public health crisis, and effective prophylactic and therapeutic agents are urgently needed. Angiotensin-converting enzyme 2 (ACE2), a carboxypeptidase degrading angiotensin II to angiotensin 1–7, has been identified as the host receptor to interact with SARS-CoV-2 spike (S) glycoprotein during viral entry^1^. Therefore, ACE2 has become a critical target for COVID-19 drug development. Human recombinant soluble ACE2 (hACE2) showed significant inhibition of SARS-CoV-2 infection in cell cultures and engineered organoids^2^. It has been tested in phase I and phase II clinical trials and revealed a good safety profile^3^. In a recent case report, hACE2 treatment in a patient with severe COVID-19 demonstrated a rapid viral clearance in plasma and respiratory system^3^. In addition, neutralizing antibodies (NAbs) directly targeting the interaction between ACE2 and receptor-binding domain (RBD) of the S protein also showed promising therapeutic effects in the clinic, and two of them got FDA EUA approval^4–6^. However, multiple mutations in the S protein have been reported^4,7–9^, which may incapacitate the current NAbs as therapeutic agents. In order to overcome viral mutant escape and to mitigate drug resistance of a specific NAb, a combination strategy is employed in which a cocktail of two or more NAbs is used^4,6^. But this approach is not only costly but also cannot ensure protection against all mutations. In this study, we used a different strategy: fusing the soluble extracellular domain of hACE2 with the Fc region of human IgG1, named HLX71, which in principle should be able to neutralize any viral mutants capable of binding to ACE2. We further investigated the activity of HLX71, and its combination with HLX70, a therapeutic NAb (described in our previous study^10^) against mutant SARS-CoV-2 pseudoviruses, live SARS-CoV-2 virus in vitro and in vivo. Our data demonstrated that the cocktail of HLX70 NAb and HLX71 fusion protein elicited synergy in antiviral activity, and exhibited a broader coverage of RBD variants.

High clearance rate and short half-life make soluble hACE2 unlikely a therapeutic agent^3,11^. hACE2 with human IgG1 Fc fusion at the C-terminus showed improved pharmacokinetics property and was able to bind to SARS-CoV and SARS-CoV-2 S protein and neutralize pseudoviruses in vitro^12^. To further evaluate its therapeutic potential, we generated the HLX71, hACE2–Fc fusion protein. ELISA results showed that HLX71 bound to SARS-CoV-2 RBD with high affinity (EC_50_ = 0.14 nM) (Supplementary Fig. S1a). HLX71 could also block the RBD protein from binding to the recombinant ACE2 protein or HEK293T cells transiently expressing hACE2, with IC_50_ of 2.56 nM and 9.8 nM, respectively (Supplementary Fig. S1b, c). Moreover, HLX71 effectively neutralized SARS-CoV-2 pseudovirus with an IC_50_ of 5.56 μg/mL (17.4 nM) and BetaCoV/Beijing/IMEBJ01/2020 live virus strain with a PRNT_50_ (50% plaque reduction neutralization test) of 1.74 μg/mL (7.83 nM) (Supplementary Fig. S1d, e). Importantly, the fusion protein remained effective in neutralizing the D614G variant virus, one of the most prevalent mutations (12.3 nM) (Supplementary Fig. S1e). In summary, HLX71 could effectively block the binding of SARS-CoV-2 to ACE2 and neutralize both SARS-CoV-2 pseudovirus and live virus infection.

Next, we sought to evaluate the in vivo efficacy of the HLX71 in hACE2-transgenic mice. A single dose of HLX71 at 15 or 50 mg/kg was given intraperitoneally 2 h after intranasal infection of SARS-CoV-2 (Fig. 1a). A lethal dose (nCoV-SH01, 4.15 × 10^5^ PFU) resulted in clinical symptoms of infection in all animals. Whereas 60% control hACE2-transgenic mice died before the scheduled euthanasia, all animals receiving HLX71 at 50 mg/kg survived (Fig. 1b), suggesting the in vivo antiviral activity of HLX71.

**Fig. 1:**
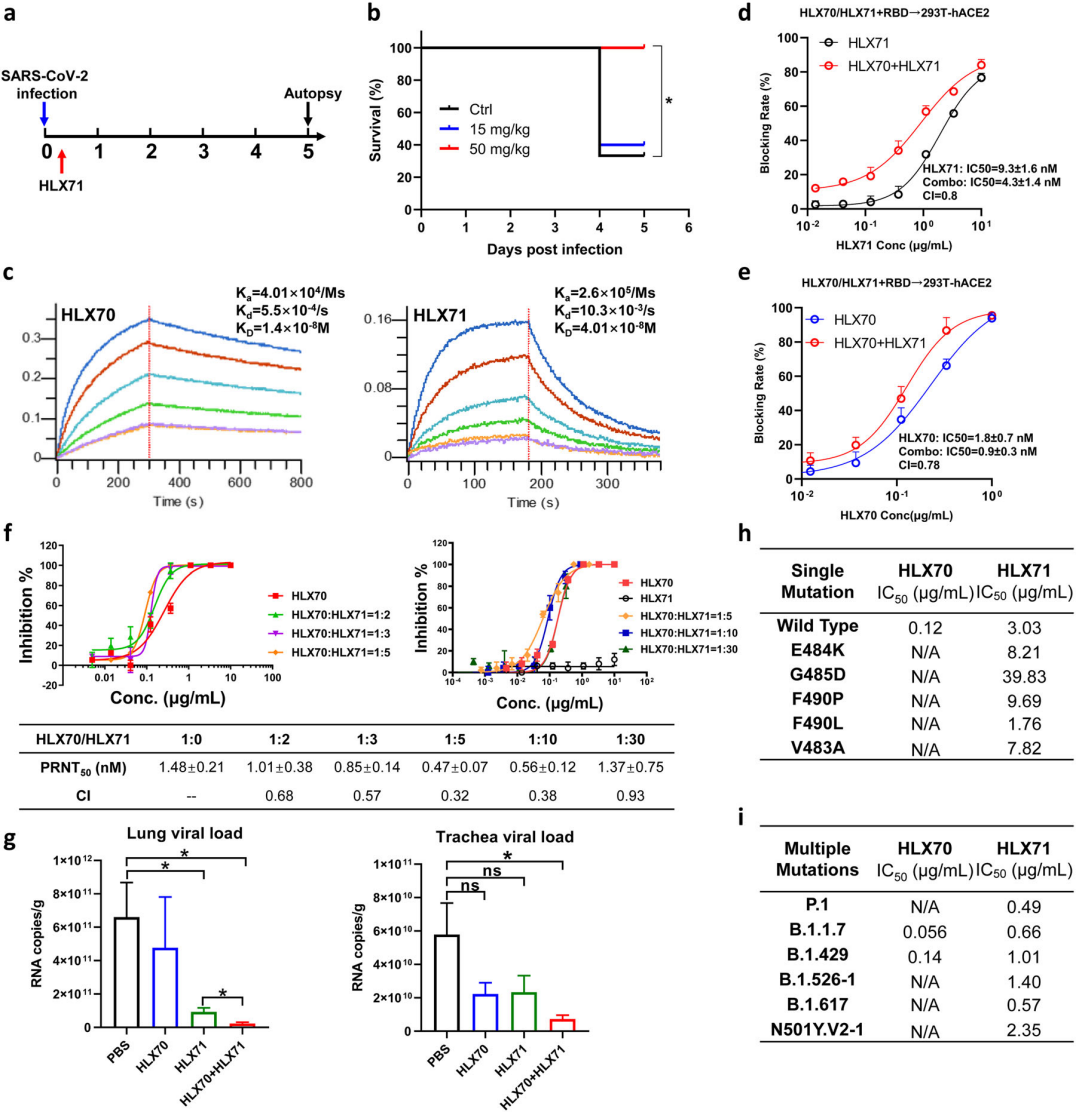
Combination of HLX70 and HLX71 increased efficacy in SARS-CoV-2 neutralization. **a** Schematic diagram for treatment schedule of HLX71 in the hACE2-transgenic mouse model. A single dose of HLX71 (15 or 50 mg/kg) or PBS control was injected intraperitoneally 2 h after the SARS-CoV-2 challenge. The mortality of the mice was recorded every day for 5 days. **b** Survival curve of SARS-CoV-2-infected mice treated with HLX71. *P < 0.05. **c** Affinity analysis of the binding of HLX70 (left) and HLX71 (right) to SARS-CoV-2 RBD. **d** RBD-blocking activity of HLX71 is enhanced by HLX70. HLX70 (0.05 μg/mL) was mixed with serially diluted HLX71 and incubated with RBD-His. The mix was then added to the 293T-hACE2 cell suspension, and an anti-His-PE antibody was used for detection. CI (combination index) values were calculated by CompuSyn software. **e** RBD-blocking activity of HLX70 is enhanced by HLX71. HLX71 (0.5 μg/mL) was mixed with serially diluted HLX70 and incubated with RBD-His. The blocking rate was measured the same way as **d**. **f** HLX70 was mixed with HLX71 at the ratio of 1:2, 1:3, 1:5, 1:10, and 1:30. Threefold serial dilutions of the mixes were added to ~500 PFU of SARS-CoV-2 virus solution, and the neutralization potency was assessed in Vero cells using PRNT. The *x* axis shows the concentrations of the HLX70. **g** In vivo efficacy of HLX70 + HLX71 combination. BALB/c mice were treated with HLX70 (*n* = 5), HLX71 (*n* = 5), or HLX70 + HLX71 combination (*n* = 5) 2 h after infection of 1.6 × 10^4^ PFU of MASCp6. Five days after the virus challenge, mice were sacrificed to analyze the virus burden in the lung and trachea. Virus titer was demonstrated as RNA copies per gram tissue. ns, not significant; *P < 0.05. **h** HLX71 neutralization activity against HLX70-resistant SARS-CoV-2 pseudovirus mutants. **i** Neutralization activity of HLX70 and HLX71 against pseudoviruses with multiple mutations.

Although HLX71 targets the conserved residues critical for ACE2–RBD binding, a cocktail of HLX71 and an NAb might be more effective in achieving broader coverage of mutants. Here, we chose P17 (code name HLX70), an NAb with strong neutralizing potency in our previous study^10^, as the combinational partner. Although the equilibrium constant (*K*_D_) is comparable, HLX70 exhibited distinct binding kinetics from HLX71, with a 6.5-fold slower association rate (*k*_a_) and an 18.7-fold slower dissociation rate (*k*_d_) (Fig. 1c), suggesting a different mode of recognition toward RBD.

The effect of blocking RBD protein from binding to the hACE2-HEK293T cells was assessed using a flow cytometry-based assay. A serially diluted HLX71 was mixed with a fixed low concentration of HLX70 (0.05 μg/mL). At this concentration, HLX70 showed no blockage of RBD. In contrast, treatment with HLX71 at increasing concentrations elicited dose-dependent antiviral effects, with an IC50 of 9.3 nM (Fig. 1d, black line). Remarkably, HLX71 plus the ineffective dose of HLX70 (0.05 μg/mL) significantly increased the RBD-blocking activity over HLX71 mono-treatment alone, with IC50 of 4.3 nM (Fig. 1d, red line). Vice versa, HLX71 could also enhance the activity of HLX70. As shown in Fig. 1d, the fusion protein HLX71 at 0.5 μg/mL elicited no RBD-blocking activity. Whereas HLX70 elicited a dose-dependent RBD-blocking activity with an IC50 of 1.8 nM (Fig. 1e, blue line), HLX70 plus an ineffective dose of HLX71 (0.5 μg/mL) shifted the dose–response curve towards the left, with an IC50 of 0.9 nM (Fig. 1e, red line). These results together suggest that the combination of HLX71 with HLX70 could synergize in blocking the binding of RBD to hACE2 on HEK293T.

To determine whether there was a synergistic antiviral effect of HLX71 and HLX70, a standard plaque reduction neutralization test (PRNT) was performed to investigate the combinational neutralization potency against SARS-CoV-2 live virus infection. The mixtures of HLX70 and HLX71 at the ratio of 1:2, 1:3, or 1:5 were serially diluted to evaluate the neutralization activity of SARS-CoV-2 (500 PFU) in Vero cells. The combination of HLX70 and HLX71 with 1:5 ratio was approximately threefold more potent than HLX70 alone (Fig. 1f, left). We repeated the neutralization assay with higher ratios of HLX70 and HLX71 (1:5, 1:10, 1:30), revealing that the ideal combination ratio remained to be 1:5 (Fig. 1f, right).

The emergence of new mutants of SARS-CoV-2 has significantly threatened the effectiveness of COVID-19 therapies^8,9^. Thus, it is urgent to develop therapies that could mitigate virus escape. We first evaluated the in vivo efficacy of the HLX70 + HLX71 combination in a mouse-adapted strain MASCp6-based model. MASCp6 contained a single N501Y mutation on the S protein^13^, which was supposed to enhance the binding affinity to mouse and human ACE2^13,14^. Compared with HLX70 or HLX71 alone, 5 mg/kg of HLX70 plus 25 mg/kg of HLX71 combination can significantly enhance virus clearance, suggesting the effectiveness of combination therapy for the new identified N501Y variant (Fig. 1g). To further evaluate the combinational effects of HLX70 and HLX71 against a broader panel of RBD variants, we mixed HLX70 and HLX71 with a ratio of 1:5 and performed the neutralization test using a pseudovirus system. We have evaluated 45 naturally occurring S variants and only a few of them (V483A, E484K, G485D, F490L, F490, P.1, B.1.526-1, B.1.617, and 501Y.V2-1) exhibited resistance to HLX70 (Fig. 1h, i and Supplementary Table S1), which were reported as the critical residues in the HLX70/RBD-binding interface^10^. Interestingly, HLX71 alone or in combination with HLX70 could effectively neutralize all 45 mutants including the HLX70-resistant variants and the widely circulated mutants (N501Y, P.1, B.1.1.7, B.1.429, B.1.526-1, B.1.617, and 501Y.V2-1) (Fig. 1i). These results confirmed our hypothesis that HLX71 has a broader coverage and the combination of HLX71 with HLX70 elicits a synergistic effect against virus infection.

In this study, we demonstrated the neutralization activity of HLX71 and that of its combination with HLX70, revealing that HLX70 and HLX71 work synergistically at very low concentrations in neutralizing SARS-CoV-2, and exhibit a broader coverage of most known viral variants, including several naturally occurring mutants that resist HLX70 alone. The combination also showed enhanced N501Y virus clearance in vivo.

HLX70, derived from the *VH3-30/VK1-39* family and recognizing both “up” and “down” states of RBD^10^, could be classified into the class 2 NAbs according to structural analyses of Barnes et al.^15^. Besides HLX70 NAb, other class 2 NAbs with similar binding epitopes, such as BD-368-2 which is under clinical trial^6^, would have combinational potential with HLX71. Further structural analyses suggested that two other classes of NAbs (class 3: S309 and class 4: CR3022) each aim at a different region^15^ and could also have functions in the combinational pair with hACE2–Fc (Supplementary Fig. S2), which needs further studies.

Besides being the receptor for SARS-CoV-2, ACE2 also serves as an RAS regulator and protects organs from Ang II-induced inflammation^3^. HLX71 contains an extracellular domain of human ACE2 (UniProt: Q9BYF1) fused with human IgG1 Fc. Thus, like ACE2, HLX71 has inherent enzymatic activity, which arouses potential concern of the side effects. However, in a recent case report, treatment with hACE2 not only reduced the viral load in the plasma and respiratory system but also resulted in a marked reduction of Ang II and other inflammatory and injury markers^3^, protecting the patient’s respiratory system. Further, treatment with HLX71 at a single dose up to 150 mg/kg did not induce any adverse effect, including cardiovascular-related abnormal in cynomolgus monkeys. Downstream signalings and Ang II-mediated effects could be explored during mono or combination therapy of targeting ACE2. Taken together, it is likely that the hACE2–Fc strategy and HLX71 could be used for combinational therapy against COVID-19, as well as against Ang II-induced inflammation.

## Supplementary information

Supplementary Information
