# Supplementary material for: hACE2 Fc-neutralization antibody cocktail provides synergistic protection against SARS-CoV-2 and its spike RBD variants
Source: Cell Discov. 2021 Jul 20;7:54. doi: 10.1038/s41421-021-00293-y (PMC8290864; doi:10.1038/s41421-021-00293-y)
Supplement: Supplementary file 1 — Supplementary Information [file 41421_2021_293_MOESM1_ESM.pdf]

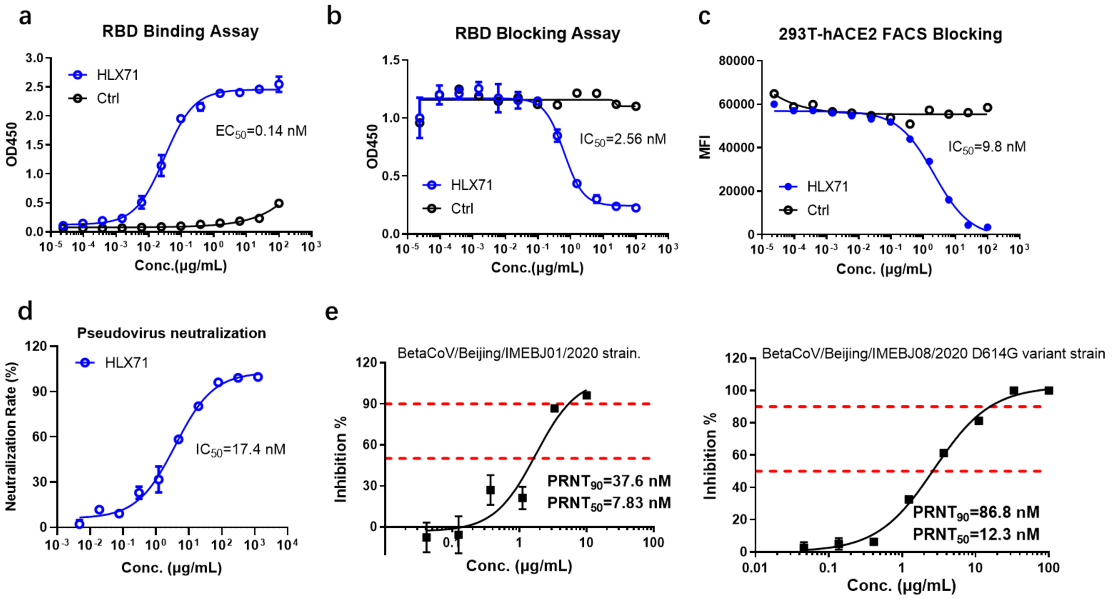

Supplementary Fig. 1, SARS-CoV-2 neutralization activity of HLX71. **a)** RBD binding assay. 96-well plates were coated with 2 μg/ml RBD protein, and then serial diluted HLX71 was added to the plates. The binding of HLX71 to RBD was detected by ELISA. **b)** Blocking of RBD-ACE2 binding by HLX71. 96-well plates were coated with 2 μg/ml RBD, incubated with 50 ng/ml ACE2-his for 1h, and then treated with serial diluted HLX71 for another 1h. The ability for HLX71 to block the SARS-CoV-2 RBD from binding to the recombinant ACE2 was measured by ELISA. **c)** HLX71 blocking of RBD binding to HEK293T cells transiently expressing hACE2. hACE2-293T cell suspension was incubated with 0.1 μg/ml RBD-Fc plus serial diluted HLX71 for 1h. RBD bound to hACE2-293T cells were detected by PE labelled anti-Fc antibody. **d)** The neutralization profile of HLX71 for SARS-CoV-2 pseudovirus. Pseudovirus were pre-incubated with serial diluted HLX71 for 1 h at 37 ℃ and then incubated with 293T-hACE2 cells for 24 h. The luminescence intensity was detected for the IC_{50} calculation. **e)** The neutralization profile of HLX71 for SARS-CoV-2 BetaCoV/Beijing/IMEBJ01/2020 or BetaCoV/Beijing/IMEBJ08/2020 strain (D614G). Then Plaque Reduction Neutralization Test (PRNT) was used to evaluate neutralization activity of HLX71 for live virus.

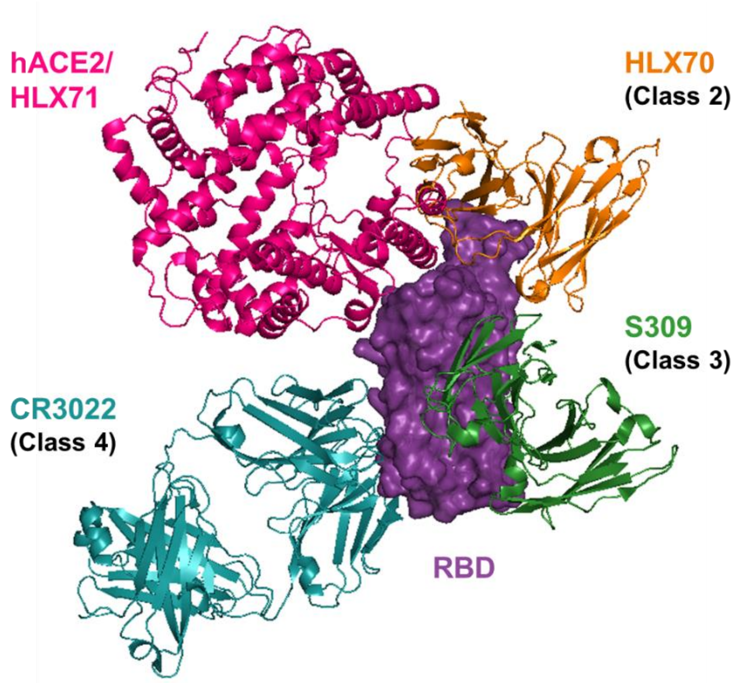

Supplementary Fig. 2, Structural analysis of HLX71 binding to RBD. The structures of the SARS-CoV-2 RBD in complex with CR3022 (PDB: 6W41) and the Spike protein in complex with S309 (PDB: 6WPS) are aligned and superimposed onto the structure of the RBD bound with hACE2/ HLX71 (PDB: 7A91). The structure of RBD with HLX70 (PDB: 7CWM) is also aligned for comparison. The classifications are based on the structural analyses of Barnes et al. HLX71 has a unique binding epitope on RBD distinct from the one bound by HLX70. Both epitopes are non-overlapping with class 3 (S309) and class 4 (CR3022) Nabs, and therefore could potentially function in a combinational pair. The structural alignments and figure were prepared using Pymol (Schrödinger, LLC).

Supplementary Table S1, Neutralization potency of HLX70, HLX71 and HLX70+HLX71 combination against pseudoparticles encoding individual escape mutants-IC_{50} summary.

Mutants	HLX70 IC_{50} (μg/mL)	HLX71 IC_{50} (μg/mL)	HLX70+HLX71* IC_{50} (μg/mL)	CI
**Wild Type**	0.1171	3.03	0.305	0.517
**K417E**	0.07108	7.722	0.204	0.500
**K444Q**	0.1126	3.511	0.398	0.684
**V445A**	0.1722	7.554	0.991	1.068
**N450D**	0.09426	6.781	0.492	0.930
**Y453F**	0.2096	1.502	0.704	0.950
**L455F**	0.1691	10.61	0.965	1.027
**E484K**	N/A	8.207	9.678	0.983
**G485D**	N/A	39.83	59.586	1.247
**F486V**	1.031	16.08	3.221	0.688
**F490P**	N/A	9.691	29.688	2.553
**Q493K**	0.5058	6.405	1.890	0.869
**D614G**	0.3599	3.484	1.126	0.791
**G339D**	0.1927	7.722	2.776	2.700
**V367F**	0.08376	0.6841	0.619	1.984
**P384S**	0.0718	3.196	0.422	1.091
**R408I**	0.1074	0.1683	1.152	7.492
**A435S**	0.04571	10.04	0.637	2.374
**S494P**	0.08575	3.802	0.8802	1.903
**N439K**	0.3153	3.598	0.8274	0.629
**G446V**	0.05772	17.76	0.743	2.181
**K458N**	0.1712	1.33	0.827	1.324
**S477I**	0.1604	6.191	0.660	0.775
**S477N**	0.2723	3.395	0.503	0.431
**F490L**	N/A	1.758	11.556	5.478
**L518I**	0.1956	8.075	0.636	0.608
**H519P**	0.1931	7.9	1.413	1.369
**T323I**	0.05941	16	1.067	3.048
**P330S**	0.2147	3.052	1.053	1.105
**A344S**	0.1991	4.061	0.539	0.562
**P384L**	0.1765	5.169	1.148	1.269
**Q414A**	0.1762	2.036	0.890	1.207
**Q414E**	0.07974	2.889	0.207	0.493
**V483A**	N/A	7.8	4.3638	0.466
**V341I**	0.1572	4.548	0.730	0.907
**K458R**	0.3519	5.924	1.145	0.703
**G476S**	0.3883	5.028	1.088	0.648
**L452R**	0.7743	2.535	1.516	0.824
**A475V**	0.5921	7.436	2.407	0.947
**N501Y**	0.1987	0.6267	0.9426	2.044
**P.1**	N/A	0.49	0.4716	0.80
**B.1.1.7**	0.056	0.66	0.31422	1.33
**B.1.429**	0.14	1.01	0.633	1.29
**B.1.526-1**	N/A	1.4	4.2372	2.52

**B.1.617**	N/A	0.57	2.2668	3.31
**N501Y.V2-1**	N/A	1.09	1.1958	0.91

* The combination of HLX70 and HLX71 is mixed with 1:5 w/w ratio.

Materials. hACE2-IRES-luc transgenic mice were purchased from Shanghai Model Organisms Center, Inc. HLX70 and HLX71 are produced by Shanghai Henlius Biotech, Inc. according to Good Manufacturing Practice guidelines. RBD of SARS-CoV-2 (DRA42) was purchased from Novoprotein. The MASCp6, SARS-CoV-2 BetaCoV/Beijing/IMEBJ01/2020 and BetaCoV/Beijing/IMEBJ08/2020 (D614G) strain were provided by State Key Laboratory of Pathogen and Biosecurity, Beijing Institute of Microbiology and Epidemiology. All the pseudovirus mutants were provided by Vazyme Biotech Co., Ltd. PE conjugated anti-his (IC050P) was purchased from R&D System, PE conjugated anti-Fc (12-4998-82) was purchased from eBioscience.

Methods

***in vivo* efficacy study.** The *in vivo* protection efficacy of HLX71 was assessed by using humanized hACE2-IRES-luc transgenic mouse model (Shanghai Model Organisms Center, Inc.). 6-8 weeks old female hACE2-IRES-luc transgenic mice were randomly divided into 3 groups and infected intranasally with a lethal dose of SARS-CoV-2 virus (nCoV-SH01, GenBank: MT121215.1). A single dose of HLX71 (15 or 50 mg/kg) or PBS control were injected intraperitoneally 2 hours after SARS-CoV-2 challenge. The mortality of the mice was recorded every day for 5 days.

For the HLX70+HLX71 *in vivo* combination study, BALB/c mice were treated with HLX70 (5 mg/kg), HLX71 (25 mg/kg) or HLX70+HLX71 (5+25 mg/kg) 2 h after 1.6×10^4 PFU of MASCp6 (mouse-adapted strain at passage 6 of SARS-CoV-2, with N501Y mutation) infection. 5 days after virus challenge mice were sacrificed to analyze the virus burden in both lung and trachea. Virus titers are demonstrated as RNA copies per gram tissue. * $p<0.05$.

BLI. Biolayer interferometry (BLI) technology was applied to study the binding affinity of HLX70/HLX71 to RBD. The Pro A sensor can specifically bind the Fc fragment of HLX70/HLX71, and HLX70/HLX71 captured on the surface of the sensor can specifically bind to RBD in solution at different concentrations (the highest used concentration is 3 μg/mL, followed by 4 times of double dilution, total 5 concentrations of RBD antigen) with association and dissociation reactions. The Octet monitors the interaction between two molecules in real time by BLI technology. Data are converted from the association and dissociation reaction between two molecules into a binding dissociation curve and are calculated as the corresponding binding constant (K_a), dissociation constant (K_d) and affinity constant or dissociation equilibrium constant (K_D) through software analysis.

HLX70+HLX71 combo blocking activity assay. 0.5 μg/mL HLX71 were mixed with serial diluted HLX70 and incubated with RBD-his for 20 min, then added into 293T-hACE2 cell suspension for 30 min incubation and PE labelled anti-his antibody was used for detection. Similarly, 0.05 μg/mL HLX70 were mixed with serial diluted HLX71 and incubated with RBD-his, then added into 293T-hACE2 cell suspension and PE labelled anti-his antibody was used for detection. All of those assays were conducted in 3 replicates.

HLX70+HLX71 combo SARS-CoV-2 neutralization activity. HLX70 was mixed with HLX71 according to the ratio of 1:2, 1:3, 1:5, 1:10 and 1:30. The mixtures were 3-fold serial diluted and added to approximately 500 PFU of SARS-CoV-2 virus solution for 1h incubation at 37 °C. Then, the mixture was added to Vero cell monolayers in a 12-well cell culture plates which were incubated in a 37 °C, 5% CO2 incubator for 1 h. After subsequent incubation, the cells were stained with crystal violet dissolved in formaldehyde to visualize the plaques. The numbers of plaques in different wells were counted and the percentage of plaque reduction was calculated as the following formula: Percent of plaque reduction (%) =100%-(plaque number with test drugs)/(plaque number without test drugs)×100%. The $PRNT_{90}$ and $PRNT_{50}$ values were determined using non-linear regression analysis (GraphPad 7.0). And CI (combination index) values were calculated by CompuSyn software. All of those assays were conducted in 3 replicates.

ELISA Binding. The binding of HLX71 to receptor binding domain (RBD) on SARS-CoV-2 S protein was investigated using an ELISA method. Recombinant SARS-CoV-2 Spike RBD-His was coated onto a 96-well plate, after incubation with serial diluted HLX71, horseradish peroxidase (HRP)-labelled Goat-anti-human-IgG-Fc-HRP was added as detection antibody.

ELISA Blocking. SARS-COV-2 RBD (Spike RBD-mFc) was incubated with various concentrations of HLX71 (4-fold serial diluted with a start concentration of 100 μg/mL) for 1 h followed by 0.5 h incubation with rhACE2-His (50 ng/mL) after washing the unbound hCAE2-Fc in the previous incubation. The blocking effect was detected by adding His-Tag antibody-HRP.

FACS Blocking. Cell-based FACS method was used to investigate the inhibitory effect of HLX71 on

RBD binding to HEK-293T-hACE2 cells (HEK-293T cells with hACE2 overexpression). Mixed recombinant SARS-COV-2 RBD (Spike RBD-Fc) with 4-fold serially diluted HLX71 or negative control were then incubated with HEK-293T-hACE2 cells for 30 min. Phycoerythrin (PE)-labelled goat anti-human IgG-Fc (PE-anti-human Fc) was used as detection antibody. The labelled cells were analyzed using FACS.

Pseudovirus neutralization activity. Wild type or different pseudovirus mutants (Vazyme DD1403-1439) were used to simulate the process of SARS-CoV-2 infecting host cells. A luciferase reporter sequence was integrated to genome of the SARS-CoV-2 pseudovirus. When the target cells are infected, the virus can express luciferase in the cells. By adding luciferase substrate (Vazyme DD1204), the relative luminescence intensity can be measured, which is correlated positively to the amount of infected pseudovirus and negatively to the neutralization activity of HLX71. Pseudovirus were pre-incubated with serial diluted HLX70, HLX71 or HLX70+HLX71 for 1 h at 37 ℃. Then 2×10^4 293T-hACE2 cells were added and incubated for 24 h. After adding luciferase substrate, luminescence intensity was detected for the IC_{50} calculation (GraphPad Prism).

HLX71 SARS-CoV-2 virus neutralization. Three-fold serially diluted HLX71 were added to approximately 100 PFU of SARS-CoV-2 virus (BetaCoV/Beijing/IMEBJ01/2020 strain or BetaCoV/Beijing/IMEBJ08/2020 D614G variant strain) solution for 1 h incubation at 37 °C. Then, the mixture was added to Vero cell monolayers in a 12-well cell culture plates which were incubated in a 37 °C, 5% CO_2 incubator for 1 h. After subsequent incubation, the cells were stained with crystal violet dissolved in formaldehyde to visualize the plaques. The numbers of plaques in different wells were counted and the percentage of plaque reduction was calculated as the following formula: Percent of plaque reduction (%) = (1-plaque number with test drug/plaque number without test drug) ×100%. The $PRNT_{90}$ and $PRNT_{50}$ values were determined using non-linear regression analysis (GraphPad 7.0).

Structural analysis. The structures of the SARS-CoV-2 RBD in complex with CR3022 (PDB: 6W41) and the Spike protein in complex with S309 (PDB: 6WPS) are aligned and superimposed onto the structure of the RBD bound with hACE2/ HLX71 (PDB: 7A91). The structure of RBD with HLX70 (PDB: 7CWM) is also aligned for comparison. The classifications are based on the structural analyses of Barnes et al. HLX71 has a unique binding epitope on RBD distinct from the one bound by HLX70. Both epitopes are non-overlapping with class 3 (S309) and class 4 (CR3022) Nabs, and therefore could potentially function in a combinational pair. The structural alignments and figure were prepared using Pymol (Schrödinger, LLC).
